# Bilateral Mandibular Fractures

**Published:** 2014-10-16

**Authors:** Srinivas M. Susarla, Edward W. Swanson, Zachary S. Peacock

**Affiliations:** ^a^Department of Plastic and Reconstructive Surgery, Johns Hopkins Hospital, Baltimore, Md; ^b^Department of Oral and Maxillofacial Surgery, Massachusetts General Hospital, Boston, Mass

**Keywords:** Facial trauma, mandibular fracture, maxillofacial injury, malocclusion, rigid fixation, intermaxillary fixation

## DESCRIPTION

A 23-year-old man presented to the emergency department following a witnessed assault. On presentation, he complained of lower facial pain, malocclusion, and numbness of the lower lip.

## QUESTIONS

1. **What are the clinical signs and symptoms of mandibular fractures?**
2. **What are the general principles for operative management of mandibular fractures?**
3. **How should teeth in the line of mandibular angle fractures be managed?**
4. **How should combined fractures of the mandibular angle and parasymphysis be managed?**

## DISCUSSION

**Figure 1:**
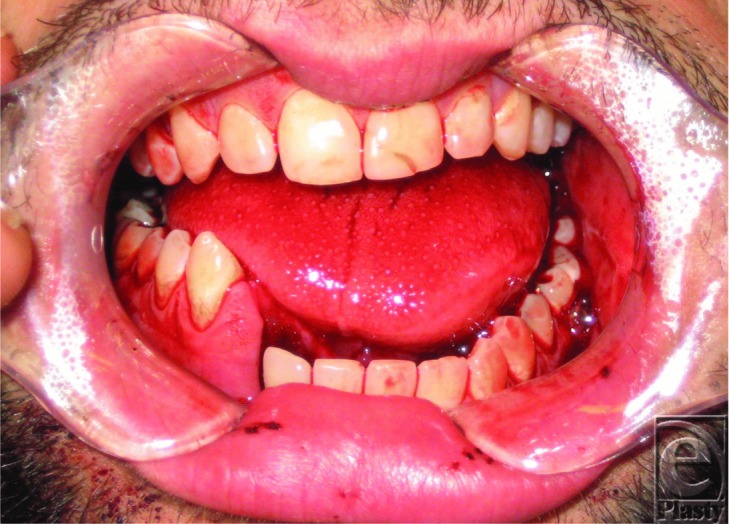
Clinical photograph demonstrating step off between the right mandibular canine and lateral incisor and general malocclusion.

**Figure 2:**
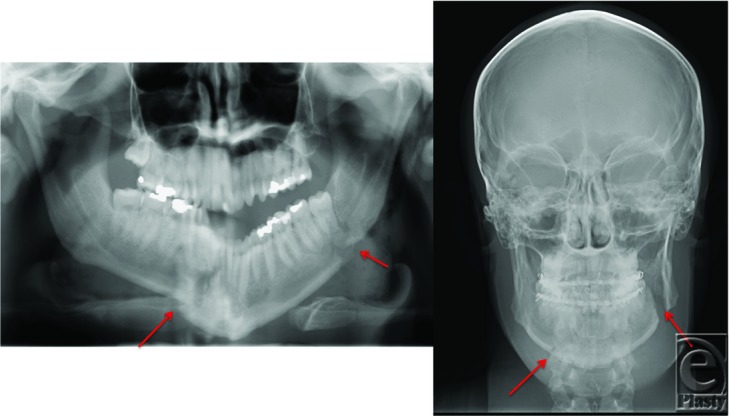
Panoramic (*left*) and anterior-posterior (*right*) radiographs demonstrating displaced right mandibular parasymphysis/body fracture and left mandibular angle fracture (*arrows*).

The most common symptoms of a mandible fracture are pain at the fracture site and subjective malocclusion.^1^^-^2 Swallowing, talking, and mouth opening/closing aggravate pain. Patients may complain of difficulty opening the jaw (trismus), loosened or fractured teeth, and lower lip numbness. Heavy intraoral bleeding may be noted at the time of injury, as well as lower facial swelling. Airway embarrassment can occur in the presence of multiple fractures, whereby the tongue insertion onto the lingual mandible is disrupted, resulting in glossoptosis. On clinical examination, the most common sign is malocclusion with a visible/palpable step off in the occlusal plane or inferior border (Fig 1). Crepitus and tenderness are noted on manipulation of the fractured segment, which is often mobile. Tenderness to palpation and increased pain with movement and compression of the adjacent mandibular segments is reported. If no fracture is believed to be present, lateral compression of both angles of the mandible should be painless with no change in the dental arch. Swelling, edema, intraoral lacerations, intraoral bleeding, loosened teeth, fractured teeth, and drooling are common. Sublingual hematoma indicates potential fractures along the lingual cortex. Decreased sensation of the lower lip often ensues from stretch of the inferior alveolar nerve at the fracture site and should be noted preoperatively. Panoramic and anteroposterior skull plain films are often sufficient for diagnosis in patients with isolated mandibular injuries (Fig 2). Axial computed tomography is a helpful adjunct for complex injuries or in patients with concomitant midface/orbital/cranial injuries.

The goals of treatment of mandibular fractures are trifold: restoration of premorbid occlusion, early return of function, and acceptable cosmesis. The basic sequence of management via open reduction requires 4 steps: restoration of premorbid occlusion, exposure of the fracture site(s), reduction of the fracture(s), and application of fixation. Restoration of the premorbid occlusion is typically done with application of intermaxillary fixation using Erich arch bars or intermaxillary fixation screws. Exposure can be done intraorally (commonly used for symphysis, parasymphysis, body and angle fractures) or extraorally (complex fractures or subcondylar injuries). Once the fractures are exposed and reduced, fixation is applied.

Management of teeth in the line of fractures remains a controversial subject. Generally, teeth in the line of fractures should be removed if they are fractured, obviously infected, or interfere with reduction of the fracture.^1^^-^2 For angle fractures, the clinician should carefully evaluate the third molar, as removal may destabilize the fracture and complicate fixation, particularly if ostectomy is required (as is often the case for impacted teeth) for removal.

More than 50% of mandibular fractures are bilateral. Combined mandibular angle and body/parasymphysis fractures should be managed with rigid fixation at the body fracture with nonrigid fixation (Champy technique) of the angle fracture.^3^^-^4 Rigid fixation of the body/parasymphysis fracture is done with an inferior border plate with a tension band (or archbar) or a reconstruction plate. Applying these forms of rigid fixation to the body fracture functionally converts a bilateral injury into a unilateral injury at the angle, which can be managed with nonrigid fixation due to the muscular forces on the mandible resulting in compression at the inferior border.

**Figure 3:**
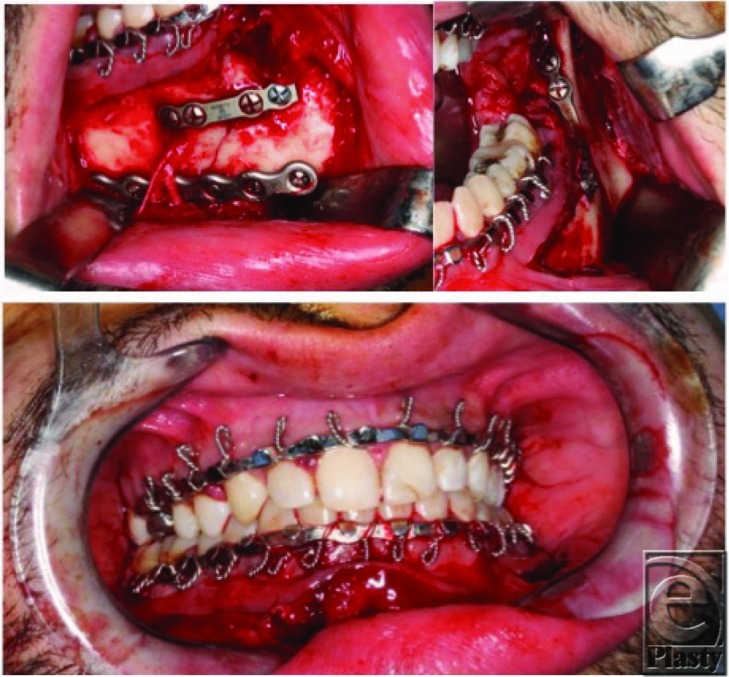
Rigid fixation applied after bony reduction of right parasymphysis/body, with preservation of mental nerve (*top left*), and superior border (ie, Champy) plate applied to left angle (*top right*). When placing screws for the tension band, care must be taken to ensure that the dental roots are not penetrated when drill holes are placed. Final occlusion (*bottom*).

**Figure 4:**
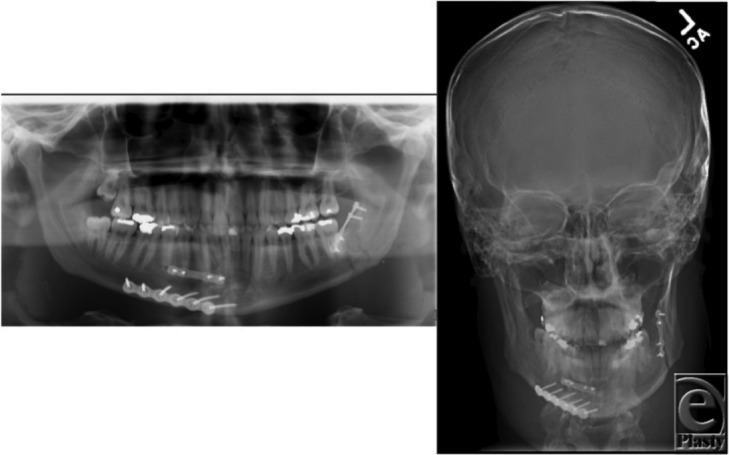
Postoperative panoramic (*left*) and anterior posterior (*right*) films, demonstrating anatomic reduction with interval removal of left mandibular third molar and application of fixation.

This patient was managed with open reduction and internal fixation at both sites. He was placed into intermaxillary fixation using Erich arch bars, and both fracture sites were exposed via intraoral vestibular approaches (Fig 3). The left mandibular third molar was interfering with bony reduction and was removed. The fractures were subsequently reduced and rigid fixation was applied to the body/parasymphysis fracture using an inferior border plate with bicortical screws and a tension band with monocortical screws. Care was taken when establishing drill holes in the mandibular body to avoid penetrating the dental roots. The left angle fracture was managed with a superior border plate bent to apply fixation in 2 planes. Postreduction films demonstrate the improved bony alignment and correction of the malocclusion (Fig 4).
